# Does the Heart Want What It Wants? A Case for Self-Adapting, Mechano-Sensitive Therapies After Infarction

**DOI:** 10.3389/fcvm.2021.705100

**Published:** 2021-09-10

**Authors:** William J. Richardson, Jesse D. Rogers, Francis G. Spinale

**Affiliations:** ^1^Department of Bioengineering, Clemson University, Clemson, SC, United States; ^2^Cardiovascular Translational Research Center, University of South Carolina School of Medicine and Columbia Veterans Affairs Health Care System, Columbia, SC, United States

**Keywords:** mechanobiology, heterogeneity, cardiac fibroblast, fibrosis, mechanotransduction, myocardial infarction

## Abstract

There is a critical need for interventions to control the development and remodeling of scar tissue after myocardial infarction. A significant hurdle to fibrosis-related therapy is presented by the complex spatial needs of the infarcted ventricle, namely that collagenous buildup is beneficial in the ischemic zone but detrimental in the border and remote zones. As a new, alternative approach, we present a case to develop self-adapting, mechano-sensitive drug targets in order to leverage local, microenvironmental mechanics to modulate a therapy's pharmacologic effect. Such approaches could provide self-tuning control to either promote fibrosis or reduce fibrosis only when and where it is beneficial to do so.

## Introduction

Roughly 800,000 myocardial infarctions (MIs) occur in the U.S. each year, and while 85% of patients survive the initial ischemic event, these survivors are left with reduced cardiac function and a shortened lifespan (1). Some emerging regenerative therapies may offer potential to restore myocardium in the collagenous infarct scar tissue (2–5), but a critical hurdle remains: how can we control the build-up of collagenous scar tissue in infarct, border zone, and remote myocardial regions post-MI? Reducing collagen content in animal infarct models has shown an ability to enhance the performance of regeneration therapies, enabling increased cell engraftment, capillary density, fractional shortening and ejection fraction (6, 7). Reducing fibrosis in remote viable myocardium post-MI also offers significant therapeutic benefit as excessive remote fibrosis is associated with mechanical and electrical cardiac dysfunction (8–10). However, reducing myocardial fibrosis must be tightly coupled to local structural integrity in order to prevent infarct scar expansion or rupture. This need is highlighted by past clinical trials of steroid treatments that decreased collagen content to disastrous effects, resulting in infarct rupture and multiple patient deaths (11). In this perspective paper, we present a case for self-adapting, mechano-sensitive therapies - i.e., therapies whose effect on fibrosis depends on the localized mechanical microenvironment and thereby reduces matrix content only when and where it is safe to do.

## Pros and Cons of Post-Infarct Fibrosis: Essential Mechanics and the Need for Adaptive Control

Myocardial fibrosis is characterized by the accumulation of extracellular matrix (ECM), which is determined by the relative balance of ECM synthesis, assembly, degradation by proteases such as matrix metalloproteinases (MMPs), and protease inhibition by enzymes such as tissue inhibitors of metalloproteinases (TIMPs). These processes are typically balanced in healthy myocardium, where a resident population of quiescent cardiac fibroblasts maintain a network of ECM within the interstitial space between cardiomyocytes providing a scaffold for cell adhesion and regulating ventricle stiffness during diastolic filling (12–14).

MI is caused by a coronary artery occlusion producing immediate ischemia and necrosis of the downstream myocardium. The dynamic wound healing process starts with inflammatory cells infiltrating the infarct zone over the first few days post-MI, releasing a variety of proteolytic enzymes to clear necrotic tissue, and signaling for the recruitment of cardiac fibroblasts via secreted cytokines (15). In the following weeks, fibroblasts migrate, proliferate, and transition to an activated phenotype with heightened contractile forces and heightened synthesis of ECM-related proteins including collagens, fibronectin, laminin, periostin, osteopontin, tenascins, thrombospondins, TIMPs, and many others (16–19). Recent work has found these activated fibroblasts can stem from not only the local resident population but also a range of different lineage sources including epithelial cells, endothelial cells, perivascular cells, fibrocytes, and bone marrow-derived progrenitors (20–22).

From a mechanical perspective, fibrosis in the infarct zone is essential for maintaining structural integrity of the scar, which becomes passive, non-contractile tissue subjected to biaxial tensile stresses from ventricular cavity pressure and remote cardiomyocyte systolic contraction. Decreased levels of collagen in the infarct scar result in reduced material stiffness and reduced thickness, which in turn drive higher wall stresses and exacerbate infarct expansion. This expansion is characterized by wall thinning in the radial direction with dilation in the circumferential-longitudinal plane, and it has long been association with reduced systolic function, increased outward bulging (dyskinesis), increased cavity dilation, increased heart failure risk, and increased wall rupture (17, 23–31). It is important to note that a “small” infarct when defined as the volume of the acutely infarcted zone relative to the uninjured myocardium is strongly correlated with much better post-MI function and clinical outcomes (32–37), so it is tempting to think that less scar tissue should be a top therapeutic objective. But numerous studies urge caution against this overly simplistic goal as drugs to reduce edema and inflammation early post-MI have repeatedly reduced collagen *density* in the infarct zone without affecting the infarct zone *size*, thereby exacerbating scar thinning and expansion as a result (38–43).

While fibrosis in the infarct zone is beneficial, excessive fibrosis in the border zone and remote zone can be detrimental to cardiac function (8–10, 44, 45). Most notably, increased ECM levels in the uninjured myocardium are associated with progressive tissue stiffening, which impairs diastolic filling (44–46). Systolic contractile function is also reduced from fibrosis-related electrical dysfunction, possibly diminished Frank-Starling effect, possibly diminished thickening from microenvironment crowding, and impaired radial thickening ability from physical coupling between the borderzone and the infarct scar (17, 28).

Collectively, data suggest that therapeutically increasing infarct stiffness and decreasing remote stiffness may help limit ventricular dilation. Further complicating the pros and cons of post-infarct fibrosis, ECM alignment, preferred orientation, and heterogeneity have demonstrated significant effects on post-MI performance (24, 26, 27, 46–49). The trade-off between pros and cons from post-infarct fibrosis should urge much caution against overly simplistic reports and conclusions in the literature. In other words, it is too simplistic to say we want to “reduce fibrosis” or “limit scar tissue” after a heart attack. We must be clearer to specify our objectives for matrix control - in what location, in what dimension, over what time period, assessed by volume vs. density, etc.? There is a critical need for more nuanced approaches for fibrotic control.

## Fibrotic Regulation: Biochemical and Mechanical Signals

Fibroblast expression of matrix-related proteins is regulated by a wide variety of biochemical agonists. Inflammatory cytokines such as tumor necrosis factor-α, interleukin 1, and interleukin 6 are upregulated immediately after injury as a stress response (50, 51) and act to suppress fibroblast activation and upregulate MMP secretion for the removal of necrotic tissue (52–55). Growth factors such as transforming growth factor β and platelet-derived growth factor are later secreted by neutrophils and macrophages as part of both an anti-inflammatory response as well as a pro-fibrotic response via upregulation of proliferation and matrix synthesis (44, 56–59). Hormonal agonists such as angiotensin II, norepinephrine, natriuretic peptides, and endothelin-1 also modulate fibroblast behavior including ECM-related gene expression (60–65).

In addition to biochemical regulation, cardiac fibroblast expression of matrix-related proteins is highly sensitive to mechanical regulation. While beating myocardium contracts with each heartbeat, infarct scar is subjected to tensile stretch, sometimes extending 5–10% in the circumferential-longitudinal dimensions (46, 47, 66). Correspondingly, cardiac fibroblasts in the infarct zone show elevated mechanotransduction signaling activity (18, 19, 67). *In vitro* mechanobiology studies have subjected fibroblasts to a variety of mechanical deformation environments including uniaxial tension, biaxial tension, and shear (68–91). Across a range of stretch magnitudes, frequencies, and durations, mechanical deformation generally produces a pro-fibrotic effect with ~2-fold increases in collagen production on average (68). In addition to substrate deformation, previous studies have shown that substrate stiffness can similarly induce mechanotransduction pathways and matrix production in cells cultured on acrylamide hydrogels, silicone-based substrates, collagen scaffolds, and other culture environments of tunable stiffness (81, 92–95). These studies have found that increased stiffnesses can induce various cell types to upregulate synthesis of matrix proteins by inducing cytoskeletal contractility and driving tension-dependent signaling pathways via inside-out integrin activation. For example, Herum et al. recently found that cardiac fibroblasts alter a host of ECM-related genes with culture on increasingly stiff substrates, including genes for collagens I and III, tenascin-c, periostin, osteopontin, thrombospondin 1, and secreted protein acidic and rich in cysteine (SPARC) (94).

Not only can mechanical force regulate fibrotic turnover via cellular mechanotransudction, force can also modulate matrix regulation in the extracellular space. A range of studies have confirmed that altered deformation of matrix proteins like collagen fibers induce altered degradation rates by proteases, presumably due to conformational changes in the matrix-protease binding pocket (68, 96–100). Other studies have shown that tension (either from cell-driven contractility or from externally applied tissue loads) can release active growth factors like TGFβ from their matrix-bound latent complexes (101, 102).

## Mechano-Adaptive Therapy: A New Strategy

Many preclinical animal studies have sought to develop effective fibrotic-related therapies for the infarcted ventricle, but there remains a striking paucity in adequate therapies. Some interventions have successfully altered matrix levels post-MI at particular time-points or spatial locations, but do so at the cost of exacerbating dysfunction at other time-points or locations. The collection of failed trials reflects the difficulty in developing therapies for the infarcted ventricle context where different spatial regions and directions require different fibrotic responses. For example, Ikeuchi et al. investigated an anti-TGFβ gene therapy in mice following left coronary ligation and found that anti-TGFβ treated mice showed lower collagen volume fraction levels and myocyte hypertrophy as well as improved clinical outcomes such as non-infarct wall thickness, left ventricular end systolic and diastolic diameters at 28 days compared to sham mice (103). However, the same population also experienced a greater degree of wall thinning 9 h post-MI with anti-TGFβ treatment, which was accompanied by heightened neutrophil invasion, inflammatory cytokine expression, and rate of mortality at 24 h, presumably caused by suppression of early reparative scar formation. This finding was later supported by Frantz et al. (104) and demonstrates the temporal nature of fibrosis, in which early scar formation can prevent further dysfunction but late fibrosis can reduce contractility, as well as the spatial nature of fibrosis, in which the infarct zone requires a robust scar formation for continued function while excess scar deposition in remote zones can lead to dysfunction.

Additional studies altering both signaling pathways and matrix-related proteins in a global manner have revealed similar challenges in temporal and spatial control. The Lindsey group has conducted several studies inhibiting specific MMPs in post-MI mouse models, finding that pharmacological inhibition of MMP9 and MMP12 reduced ejection fractions compared to saline-treated controls, with anti-MMP9 treatment increasing leukocyte infiltration and both treatments reducing apoptosis at 1 week post-MI (105, 106). Interestingly, a knockout mouse model of TIMP3 also increased complications related to early ventricular wall thinning, with TIMP3^−/−^ mice demonstrating lower hydroxyproline content, procollagen synthesis, and TGFβ expression 2 days post-MI as well as a 4-fold increase in cardiac rupture compared to wild-type mice (107). The increases in early complications after MI for seemingly opposite modes of treatment suggest that global inhibition of one single pathway or mechanism may produce counterintuitive effects in overall tissue remodeling.

**Figure 1:**
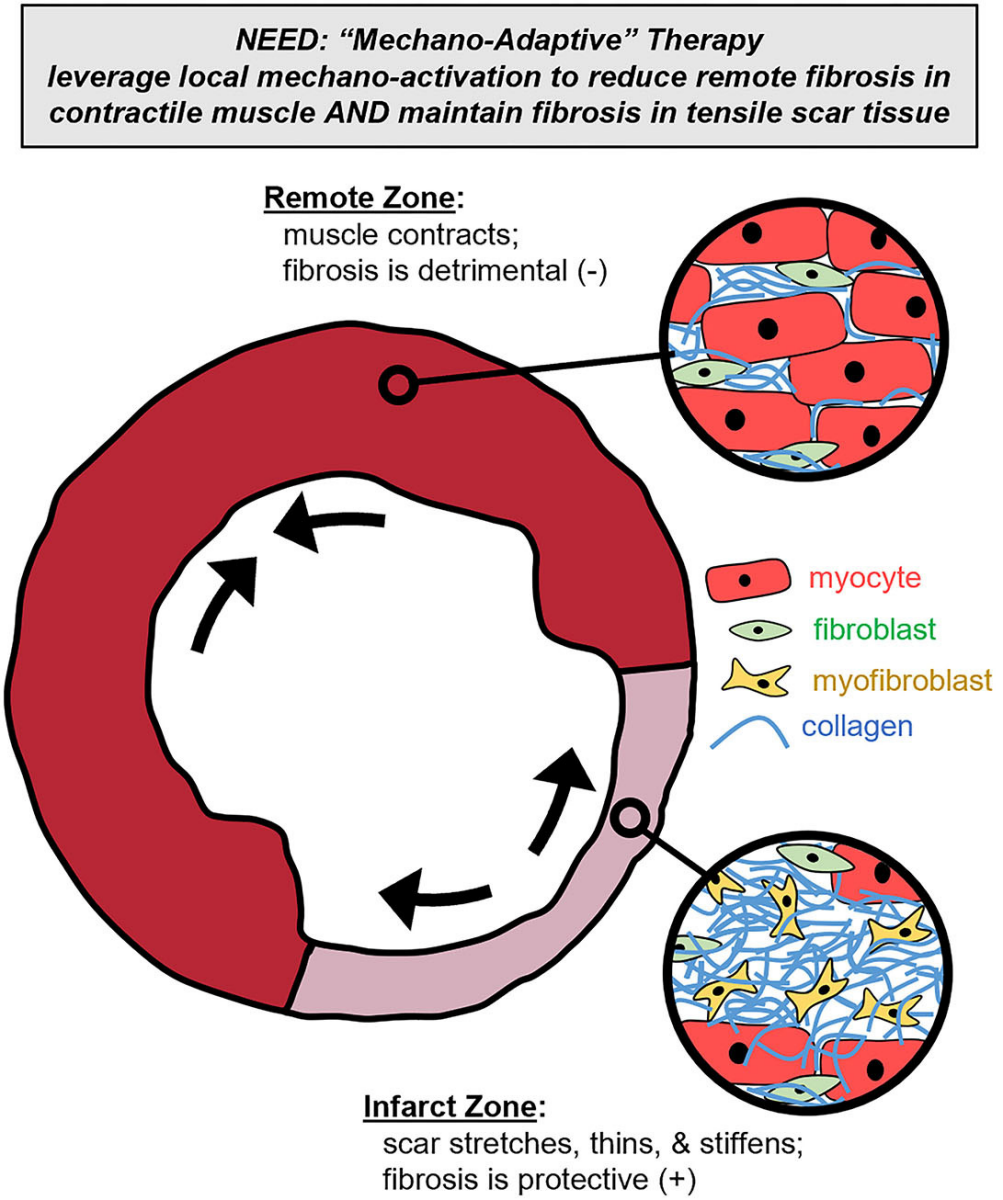
Mechano-adaptive therapy. To meet the spatially complex needs of the left ventricle cross-section post-infarction, we propose that localized mechanical cues could be leveraged to modulate a therapy's pharmacologic effect. Specifically, such a therapy could reduce collagen content in contractile muscle subregions (where fibrosis is detrimental) while simultaneously maintaining collagen content in the stretching and stiffening scar subregions (where fibrosis is beneficial).

It is increasingly clear that improving post-MI fibrotic control will require therapeutic interventions producing spatially distinct responses in order to reduce matrix buildup in remote and regenerating contractile zones while maintaining or increasing matrix buildup in passive scar zones. As a new, alternative fibrotic intervention strategy, we propose that local mechanics can provide self-adapting feedback to modulate collagen signaling and thereby enable self-adjusting specificity for anti-fibrotic therapy (Figure 1). Localized deformation and stiffness depend on local tissue structure: stiff, collagenous infarct scar stretches while functioning remote muscle contracts, with intermediate stretch levels in the infarct border zone (24, 108–111). The sensitivity of fibroblasts to local mechanical stretch presents an opportunity to design therapies that reduce fibrosis in areas that don't need it (i.e., remote or regenerating myocardium that undergoes repeated contraction) while maintaining or increasing collagen in areas that do need it (i.e., stiffening scar tissue that undergoes persistent stretch). Such a therapy could provide spatial, temporal, and even patient-specific adaptability by tailoring its effect to the localized mechanical need (112).

The premise of mechano-adaptive therapy is supported by the extensive connection between intracellular mechanotransduction signaling pathways and chemotransduction signaling pathways. Studies investigating biochemical and mechanical signals have identified evidence of crosstalk between modes of signaling, both through common intermediate pathways as well as through secondary activation of one or more additional pathways. Examples of common intermediates include angiotensin II type 1 receptors, which have been shown to mediate cardiac fibroblast gene expression in response to both increased stiffness and interstitial fluid flow (113, 114), and phosphoinositide 3-kinase/Akt signaling, which in addition to regulating TGFβ and TNFα signal transduction (115–117) has been shown to function downstream of β1-integrin signaling to regulate fibroblast apoptosis (118). Several groups have observed the secondary activation of biochemical signaling mechanisms initiated by biomechanical signaling and vice versa. The Hinz group has investigated the activation of TGFβ by fibroblasts in response to mechanical stimuli for example finding that fibroblast-generated forces can release latent TGFβ from the surrounding ECM via β-integrins, thereby inducing a positive feedback loop for further activation (102, 119). This feedback could be amplified by other cellular sources of TGFβ such as macrophages, which are concentrated in the infarcted zone during the inflammatory response to ischemia and thereby influence the local fibrotic niche (120–122). Conversely, canonical transient receptor potential (TRPC) channels have been appreciated for their role as stretch-activated ion channels in promoting cardiac fibroblast activation (67), but Davis et al. observed that the TRPC6 channel was required for TGFβ-induced activation of fibroblasts (123). TRPC6^−/−^ cells displayed attenuated αSMA fiber incorporation and gel contraction in dermal fibroblasts with TGFβ treatment as well as significantly smaller scar size and higher rates of ventricular rupture in a post-MI mouse model, demonstrating an additional mechanism-of-action beyond canonical signaling via Smad signaling.

Many studies have now shown that mechano-chemo-signaling pathway interactions ultimately give rise to mechano-sensitive gene expression responses of cells stimulated by chemical agonists (70, 79, 84, 90, 124–126). In other words, the presence of mechanical stimulation can amplify, dampen, or even reverse the effect of biochemical stimulation. Spatial differences in mechanical cues such as cyclic tension and tissue stiffness observed in the post-MI environment act as local mediators of fibroblast activation compared to remote myocardium, and so it is expected that global pharmacological treatments would exert differential responses on infarct- and remote-localized fibroblasts. Indeed, Ramirez and colleagues observed that in a post-MI mouse model, pharmacological treatments with valsartan (an angiotensin receptor blocker) and aliskiren (a renin inhibitor) primarily affected cardiac gene expression of ECM-related proteins in remote regions with minimal changes in infarcted myocardium (127). We suspect that, in similar fashion, mechano-sensitive therapies could be identified to remove detrimental fibrosis in the remote zone while simultaneously enhancing beneficial fibrosis in the infarct zone. It is also possible, though currently unknown, that cardiac fibroblasts arising from different lineages across the infarcted vs. remote zones could demonstrate region-specific sensitivities to mechano-chemo-stimuli and thereby amplify region-specific therapy responses.

Given the potential capabilities of mechano-adaptive therapies, a pressing question remains: how do we prospectively design such perturbations to leverage local mechanics for spatially and temporally adaptive benefit? One possible approach is the continued advance of higher-throughput tissue culture platforms that provide *in vitro* screening tools for drug discovery within increasingly physiologic environments. Culture arrays that can modulate the local mechanical environment can help identify pharmacologic perturbations whose effects of matrix turnover are mechano-adaptive (84, 128–132).

Yet even with high-throughput experimental approaches, the complex fibrotic regulatory network presents hundreds of potential pharmacologic targets, and millions of potential target combinations whose combined effects may not be intuitive. This challenge has motivated the advance of computational modeling approaches to predict influential mechanisms-of-action and cellular behavior while accounting for complex conditions and non-linear network dynamics (133–135). Computational models of molecular and cellular systems using mechanistic models, statistical models, and artificial intelligence approaches have been valuable in drug discovery and regulatory approval, both from academic and industry perspectives (136). In recent work, we and others have developed large-scale network models of mechanotransduction signaling capable of computationally predicting how various pathways are activated or inhibited by mechanical stimuli, how these pathways interact with other biochemical stimuli, and how these pathways alter downstream gene expression related to fibrotic turnover (137–141). Zeigler and colleagues, for example, demonstrated sizable cross-talk between TGFβ and biomechanical signaling in a model of cardiac fibroblast signaling, as both model predictions and experimental validation found that the TGFβ1 receptor is necessary for mechanically-induced αSMA expression and contraction of collagen gels (137). The same model was also used to simulate cellular responses to time courses of post-MI biochemical stimuli mimicking the inflammatory, reparative, and maturation phases of wound healing (142). The authors found that different intracellular signaling species mediate collagen I and III expression during early, intermediate, and late phases of wound healing, thus informing potential time courses for treating MI patients with anti-fibrotic therapeutics. In other work, Tan and colleagues demonstrated the potential for network models to enable comprehensive drug screens *in silico* by predicting cell responses to knockdowns of individual or combinations of signaling species (138). They used a model of cardiomyocyte mechanotransduction to identify several perturbations in combination with angiotensin receptor blockers that could provide therapeutic benefit above the use of angiotensin receptor blockers alone, such as those inhibiting ET-1 receptors or integrins. Applications of this computational approach to the spatially varying post-MI microenvironments can provide a basis for further experimental studies and ultimately improve the efficiency of drug discovery by accounting for the myriad of interconnected signaling pathways between mechano- and chemo-transduction.

## Conclusion

Does the heart want what it wants? That is to say, given the complex infarcted ventricle where fibrosis can be both beneficial and detrimental depending on the location, timing, and direction, can we design post-MI therapy that lets the heart's own local mechanical environment dictate the therapeutic effects according to the heart's own personalized, spatial, and temporal needs. While chemo-mechano-interactions complicate the regulatory network driving fibrotic turnover, we have proposed that localized mechanics also offer an exciting opportunity for tailoring fibroblast behavior to localized mechanical cues. Mechano-adaptive therapies could thereby provide self-adapting responses and modulate matrix only when and where it is beneficial to do so.

## Data Availability Statement

The original contributions presented in the study are included in the article/supplementary material, further inquiries can be directed to the corresponding author/s.

## Author Contributions

All authors contributed substantively to this work including idea generation, review of past studies, text writing, and text editing.

## Funding

The authors gratefully acknowledge funding from the National Institutes of Health (HL144927 and GM121342) and the American Heart Association (17SDG33410658).

## Conflict of Interest

The authors declare that the research was conducted in the absence of any commercial or financial relationships that could be construed as a potential conflict of interest.

## Publisher's Note

All claims expressed in this article are solely those of the authors and do not necessarily represent those of their affiliated organizations, or those of the publisher, the editors and the reviewers. Any product that may be evaluated in this article, or claim that may be made by its manufacturer, is not guaranteed or endorsed by the publisher.
